# Targeting the p53-MDM2 interaction by the small-molecule MDM2 antagonist Nutlin-3a: a new challenged target therapy in adult Philadelphia positive acute lymphoblastic leukemia patients

**DOI:** 10.18632/oncotarget.7339

**Published:** 2016-02-12

**Authors:** Stefania Trino, Ilaria Iacobucci, Daniela Erriquez, Ilaria Laurenzana, Luciana De Luca, Anna Ferrari, Andrea Ghelli Luserna Di Rorà, Cristina Papayannidis, Enrico Derenzini, Giorgia Simonetti, Annalisa Lonetti, Claudia Venturi, Federica Cattina, Emanuela Ottaviani, Maria Chiara Abbenante, Domenico Russo, Giovanni Perini, Pellegrino Musto, Giovanni Martinelli

**Affiliations:** ^1^ Laboratory of Pre-Clinical and Translational Research, IRCCS Referral Cancer Center of Basilicata, Rionero in Vulture (PZ), Italy; ^2^ Department of Experimental, Diagnostic and Specialty Medicine, Institute of Hematology “L. and A. Seràgnoli”, University of Bologna, Bologna, Italy; ^3^ Department of Pharmacy and Biotechnology, University of Bologna, Bologna, Italy; ^4^ Department of Biomedical and Neuromotor Sciences, University of Bologna, Bologna, Italy; ^5^ Chair of Hematology and BMT Unit, University of Brescia, Brescia, Italy; ^6^ Scientific Direction, IRCCS Referral Cancer Center of Basilicata, Rionero in Vulture (PZ), Italy

**Keywords:** acute lymphoblastic leukemia, p53, MDM2, Nutlin-3a

## Abstract

MDM2 is an important negative regulator of p53 tumor suppressor. In this study, we sought to investigate the preclinical activity of the MDM2 antagonist, Nutlin-3a, in Philadelphia positive (Ph^+^) and negative (Ph^−^) leukemic cell line models, and primary B-acute lymphoblastic leukemia (ALL) patient samples. We demonstrated that Nutlin-3a treatment reduced viability and induced p53-mediated apoptosis in ALL cells with wild-type p53 protein, in a time and dose-dependent manner, resulting in the increased expression of pro-apoptotic proteins and key regulators of cell cycle arrest. The dose-dependent reduction in cell viability was confirmed in primary blast cells from B-ALL patients, including Ph^+^ ALL resistant patients carrying the T315I *BCR-ABL1* mutation. Our findings provide a strong rational for further clinical investigation of Nutlin-3a in Ph^+^ and Ph^−^ ALL.

## INTRODUCTION

Acute lymphoblastic leukemia (ALL) is a malignant tumor of hematopoietic precursors committed to the B- or T-cell lineage. ALL is the most common childhood hematopoietic tumor; it is less common in adult people and generally carries a worse prognosis. The most frequent genetic abnormality associated with adult ALL patients is the t(9:22) translocation, also called Philadelphia chromosome (Ph) translocation [1, 2]. Despite recent advances in the treatment of adult and, especially, of childhood ALL, several disease subtypes still have a poor outcome and relapse due to the failure of current therapies caused by drug resistance or toxicity events [3, 4]. Therefore, novel targeted therapies are needed to improve the outcome of ALL patients.

**Figure 1:**
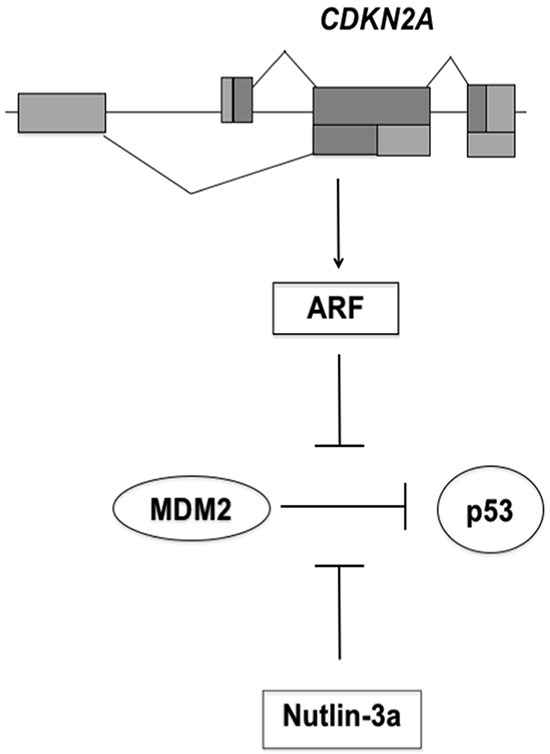
Schematic model for p53 activation by Nutlin-3a The *CDKN2A* locus encodes ARF protein that binds MDM2. This interaction antagonizes the ubiquitin ligase activity of MDM2, stabilizes p53 and triggers p53 signaling. *CDKN2A* deletion eliminates the tumor surveillance mechanism based on ARF-MDM2 interaction. Nutlin-3a binds MDM2 with consequently activation of p53 pathway.

P53 is a tumor suppressor protein with a key role in the maintenance of genetic stability and in prevention of cancer development [5]. In unperturbed cells the activity of p53 is finely regulated by MDM2 (murine double minute 2). This protein binds the N-terminal domain of p53 and inhibits its transcriptional activity. In addition, MDM2 has an E3 ubiquitin ligase activity that targets p53 to proteasomal degradation [5-7]. ARF, an alternate reading frame protein encoded by the tumor suppressor gene *CDKN2A*, participates to the regulation of the p53 pathway by interacting with MDM2. ARF binds to MDM2 and prevents the ubiquitination of p53 (Figure 1), thereby stabilizing it [8]. Disruption of the p53 pathway is strongly associated with tumorigenesis. The *TP53* gene is inactivated in 50% of human tumors by deletion or mutations that impair its DNA binding and transactivation activity [9, 10].

Different studies revealed that *TP53* mutations were rather infrequent in ALL, but they analyzed a small cohort of patients and mainly childhood or relapsed cases [11-13]. Recently, Stengel et al. demonstrated a *TP53* mutation incidence of 15.7% in a large cohort of B- and T-ALL patients [14]. Moreover, most cases of ALL expressed wild-type *TP53* but the protein does not function properly due to overexpression of *MDM2* [15] and to deletion of *CDKN2A* gene [16, 17].

Previous studies, by Vassilev and colleagues, identified the first potent and selective small-molecule MDM2 antagonists, the Nutlins [18]. These cis-imidazoline compounds compete with MDM2 for p53 binding, thus preventing the formation of the p53-MDM2 complex and the negative regulation of p53 (Figure 1) [19]. Nutlins have been shown to inhibit the p53-MDM2 interaction in different cell types with a high specificity, leading to p53 stabilization and activation of p53 pathway, resulting in apoptosis or quiescence [18, 19]. Moreover, as a consequence of nutlin treatment, p53 may prevent cellular senescence, inhibiting mTOR pathway [20, 21].

It has been previously demonstrated that Nutlin-3a induces apoptosis in pediatric ALL with wild-type *TP53* and over-expression of *MDM2* [22], and that inhibition of PI3K/AKT pathway synergized the ability of Nutlin-3a to induce apoptosis in a set of ALL cell lines [23]. Kaindl U. et al. also reported that co-exposure of Nutlin-3a and chemotherapeutic drugs reduced cell viability and potentiated apoptosis in childhood ALL cell lines with ETV6/RUNX1 fusion gene [24]. However, Nutlin-3a effects are still not completely elucidated in adult B-ALL. Thus, in the present study we investigated the therapeutic potential of p53 activation by Nutlin-3a in Ph^+^ and Ph^−^ ALL cell lines and primary cells from adult B-ALL.

## RESULTS

### MDM2 inhibition reduces viability of Ph^+^ and Ph^−^ leukemia cell lines and primary ALL cells

**Figure 2:**
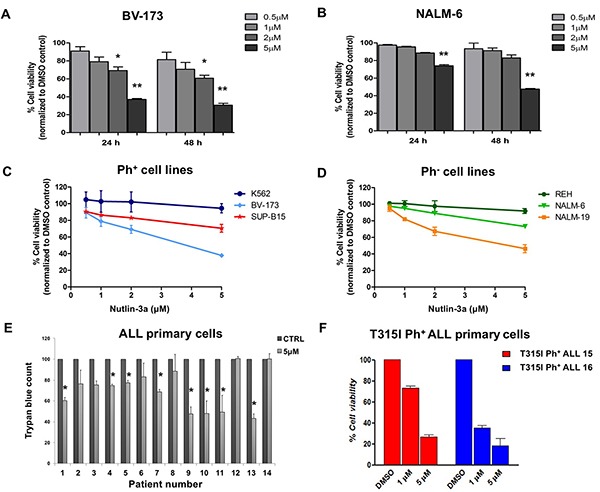
Cell viability reduction in ALL cell lines after Nutlin-3a treatment **A.** BV-173 and **B.** NALM-6 viability was evaluated by MTS test after treatment with increasing concentrations of Nutlin-3a (0.5 μM, 1 μM, 2 μM, 5 μM) at 24 and 48 hours. Results are expressed as percent viability relative to DMSO-treated controls. The bar-graphs represent mean with S.D. from three independent experiments. Viability of **C.** Ph^+^ (BV-173, SUP-B15 and K562) and **D.** Ph^−^ (REH, NALM-6 and NALM-19) leukemic cell lines was evaluated by MTS test after treatment with increasing concentrations of Nutlin-3a treatment (0.5 μM, 1 μM, 2 μM, 5 μM) at 24 hours. **E.** Trypan blue count was performed in primary cells, isolated from 9 Ph^+^ (UPN 1-9) and 5 Ph^−^ (UPN 10-14) ALL patients after 24 hours of Nutlin-3a treatment at 5 μM concentration (or DMSO-control). **F.** Viability of mononuclear cells isolated from 2 ALL patients (UPN 15-16) harboring T315I mutation was evaluated by MTS test after 24 hours of Nutlin-3a treatment at 1 μM and 5 μM concentrations. Results are expressed as percent viability relative to DMSO-treated controls. The bar-graphs represent mean with S.D. Statistically significant analyses are indicated by asterisks: *p<0.05, **p<0.01.

In order to investigate the effects of Nutlin-3a on ALL cells, we firstly analyzed cell viability of Ph^+^ and Ph^−^ leukemic cell lines treated with increasing drug concentrations at different time points. The active Nutlin-3a enantiomer significantly reduced cell viability in BV-173 Ph^+^ cells (Figure 2A) in dose dependent manner (p<0.05 and p<0.01 at 2 μM and 5 μM, respectively) and in NALM-6 Ph^−^ cells (Figure 2B) in a dose- and time-dependent manner (p<0.01 at 5 μM) at 24 and 48 hours after treatment.

Numerous preclinical and clinical studies reported that the activation of p53 signaling, mediated by small molecule MDM2 inhibitors such as Nutlins, was dependent on the wild-type status of p53 [19, 25]. Therefore, we tested leukemic cell lines with wild-type, mutated or null p53 for sensitivity to Nutlin-3a treatment. Nutlin-3a efficiently inhibited the growth of Ph^+^ and Ph^−^ ALL cells with wild-type p53 (Figure 2C and 2D, respectively). In contrast, no significant changes in cell viability were observed in p53-null chronic myeloid leukemia (CML) cell line K562 (Figure 2C) and in Ph^−^ p53-mutated ALL cell line REH (Figure 2D) after incubation with MDM2 inhibitor.

We then analyzed the effect of Nutlin-3a treatment on the viability of primary Ph^+^ and Ph^−^ ALL cells carrying wild-type p53, by incubating cells with 5 μM Nutlin-3a for 24 hours. Nutlin-3a induced a significant reduction of viability in 5 Ph^+^ and 3 Ph^−^ ALL primary cells compared to their untreated counterpart (p<0.05) (Figure 2E). Notably, the dose-dependent reduction in cell viability was confirmed in primary blast cells from two Ph^+^ ALL patients with the T315I *BCR-ABL1* kinase domain mutation, which is responsible for resistance to currently available TKIs (Figure 2F).

### MDM2 inhibitor activates p53 pathway in ALL cells with wild-type p53

**Figure 3:**
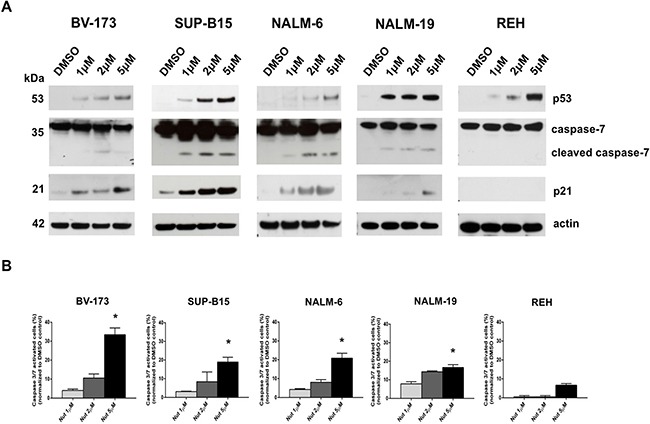
Activation of p53-downstream pathway by MDM2 inhibition in ALL cells with wild-type p53 **A.** Western blot analysis of p53, caspase-7, p21 and actin was performed in BV-173, SUP-B15, NALM-6, NALM-19 and REH cell lines after treatment with increasing concentrations of Nutlin-3a (1 μM, 2 μM, 5 μM) or DMSO control (0.1%) at 24 hours. **B.** Caspase 3/7 activity assay was performed in BV-173, SUP-B15, NALM-6, NALM-19 and REH cell lines after treatment with increasing concentrations of Nutlin-3a (1 μM, 2 μM, 5 μM) or DMSO control (0.1%) at 24 hours. Statistically significant analyses are indicated by asterisks: *p<0.05.

To investigate the effect of MDM2 inhibitor on the p53 pathway, we analyzed the expression of p53 and its target genes in ALL cell lines. As expected, western blot analysis revealed an increased p53 protein level in the p53 wild-type cell lines BV-173, SUP-B15, NALM-6 and NALM-19 cells compared to the untreated control (Figure 3A). In addition, the activation of the p53 pathway was demonstrated by increased expression of p53 downstream target, p21, and activation of the cleaved caspase-7 apoptotic marker. Furthermore, Nutlin-3a exposure increased p53 protein levels in p53-mutated REH cells, but the protein was functionally inactivated; thus, no activation of p53 pathway was observed (Figure 3A). Caspase 3/7 signaling was also detected by cytometric analysis showing a significant increase of caspase 3/7 activation in BV-173, SUP-B15, NALM-6 and NALM-19 cells (p<0.05) (Figure 3B) after treatment with 5 μM of Nutlin-3a but not in REH cells.

### Inhibition of MDM2 induces apoptosis in Ph^+^ and Ph^−^ leukemia cell lines

**Figure 4:**
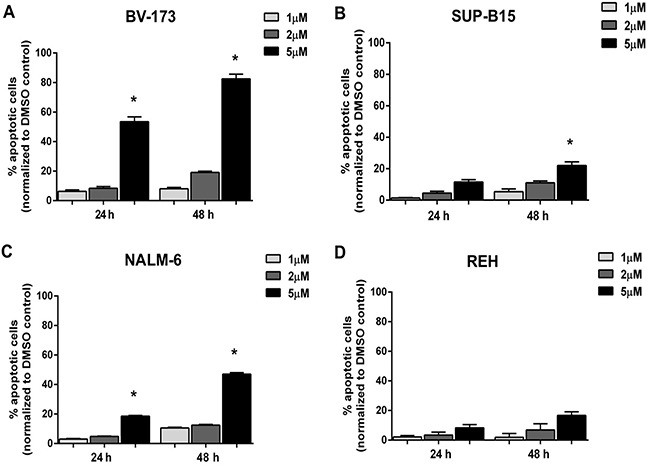
Induction of apoptosis in ALL cells after Nutlin-3a treatment Apoptosis induction was evaluated in **A.** BV-173, **B.** SUP-B15, **C.** NALM-6 and **D.** REH cell lines by Annexin V test after 24 and 48 hours of Nutlin-3a treatment at increasing concentrations (1 μM, 2 μM, 5 μM). Results are expressed as percent apoptosis relative to DMSO-treated controls. The bar-graphs represent mean with S.D. from three independent experiments. Statistically significant analyses are indicated by asterisk: *p<0.05.

To better characterize the reduction of cell viability induced by Nutlin-3a treatment in ALL cells, we determined the percentage of apoptotic cells after MDM2 inhibitor treatment in ALL cell lines. Our data showed that Nutlin-3a significantly induced a dose- and time-dependent apoptosis in BV-173 (from 6% to 53% at 24 hours and from 8% to 82% at 48 hours) (Figure 4A), SUP-B15 (from 2% to 12% at 24 hours and from 5% to 20% at 48 hours) (Figure 4B) and NALM-6 (from 3% to 20% at 24 hours and from 10% to 48% at 48 hours) (Figure 4C) ALL cell lines with wild-type p53, using 1 μM, 2 μM and 5 μM of Nutlin-3a. By contrast, REH cells, that harbored p53 mutation, did not show significant differences in viability after Nutlin-3a treatment (Figure 4D).

### Nutlin-3a enhances cell viability reduction in Ph^+^ ALL cell lines in combination with TKIs

**Figure 5:**
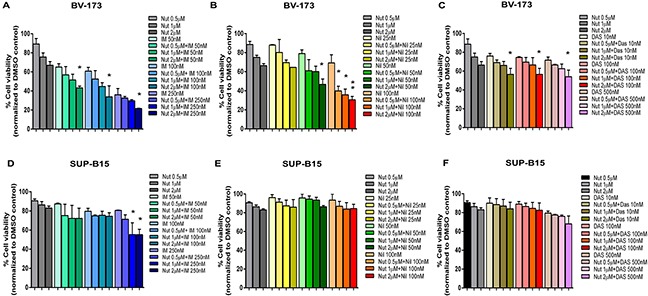
Effect of MDM2 inhibitor and TKIs combination on ALL cell lines **A, B, C.** BV-173 and **D, E, F.** SUP-B15 viability was evaluated by MTS test after 24 hours of Nutlin-3a (Nut) treatment (0.5 μM, 1 μM, 2 μM) alone or in combination with (A, D) Imatinib (IM) (50 nM, 100 nM, 250 nM) or (B, E) Nilotinib (Nil) (25 nM, 50 nM, 100 nM) or (C, F) Dasatinib (Das) (10 nM, 100 nM, 500 nM). Results are expressed as percent viability relative to DMSO-treated controls. The bar-graphs represent mean with SD from three independent experiments. Statistically significant analyses are indicated by asterisks: *p<0.05, **p<0.01.

We exposed BV-173 and SUP-B15 cell lines to increased concentrations of Nutlin-3a in combination with the TKIs Imatinib, Nilotinib or Dasatinib. The combination of Nutlin-3a and Imatinib, Nilotinib or Dasatinib significantly reduced viability of BV-173 cells in a dose dependent manner after 24 hours of treatment, compared with single TKI treatment (Figure 5A, 5B, 5C). In SUP-B15 the co-exposure of Nutlin-3a and TKIs is less effective (Figure 5D, 5E, 5F); in fact, only the combination of Nutlin-3a and Imatinib caused a significant reduction of viability, as shown in Figure 5D. Moreover, since SUP-B15 cell line was resistant to Nilotinib treatment, the combination of Nutlin-3a and Nilotinib (Figure 5E) had the same effect of Nutlin-3a alone on cell viability.

### Gene expression signature is associated with response to MDM2 inhibitor

**Figure 6:**
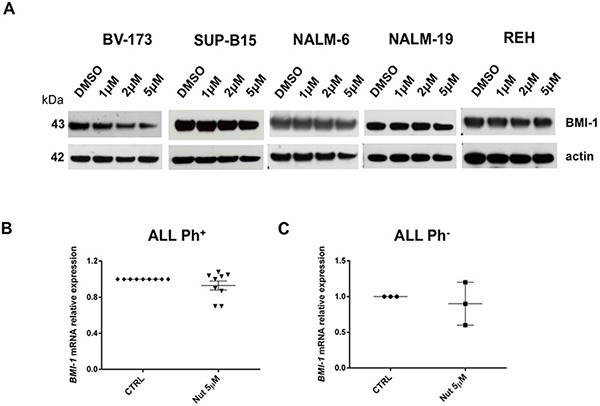
Gene expression signature associated with Nutlin-3a treatment **A.** Western blot analysis of BMI-1 and actin was performed in BV-173, SUP-B15, NALM-6, NALM-19 and REH cell lines after 24 hours treatment with increasing concentrations of Nutlin-3a (1 μM, 2 μM, 5 μM) and with DMSO control. *BMI-1* mRNA relative expression in primary cells, isolated from **B.** 9 Ph^+^ (UPN: 1-9) and **C.** 3 Ph^−^ ALL patients (UPN: 10-12), was determined by qRT-PCR assay after 24 hours of 5 μM Nutlin-3a (Nut) treatment.

To elucidate the molecular consequences of p53 activation upon Nutlin-3a exposure in ALL cells and to identify novel potential biomarkers of clinical activity, we performed gene expression profile analysis of the Nutlin-3a sensitive cell lines BV-173 and SUP-B15 after 24 hours of drug exposure and compared them with DMSO-treated cells (DMSO 0.1%). A total of 621 genes (48% down-regulated versus 52% up-regulated) were differentially expressed (p<0.05). They included genes involved in cell cycle and apoptosis control (e.g. *Histone H1, TOP2, GAS41, H2AFZ*), in the down-regulation of Hedgehog signaling (e.g. *BMI-1, BMP7, CDKN1C, POU3F1, CTNNB1, PTCH2*) and stemness genes, as well as in the down-regulation of genes with a role in the inhibition of *INK4/ARF*. Both *GAS41* (growth-arrest specific 1 gene) and *BMI1* (a polycomb ring-finger oncogene) are repressors of *INK4/ARF* and *p21* genes and their aberrant expression contributes to stemness maintenance in malignant cells [26, 27]. Moreover, the reduction of BMI-1 protein levels was associated with apoptosis in tumor cells and increased susceptibility to cytotoxic agents and radiation therapy [28]. *BMI-1* and *GAS41* were down-regulated after *in vitro* treatment with Nutlin-3a (fold-change: −1.10 and −1.35, respectively; p-value 0.03 and 0.01, respectively) (Table 1). Since BMI-1 has a crucial role in the control of apoptosis, we validated microarray data by Western blot analysis. BMI-1 protein expression decreased in BV-173 cells after Nutlin-3a treatment (Figure 6A), while they remained constant in the others Ph^+^ and Ph^−^ cell lines (Figure 6A).

**Table 1:** The table reported differentially expressed genes between control (DMSO) and BV-173 and SUP-B15 sensitive cells treated with 2 μM of Nutlin-3a for 24 hours

Gene Symbol	Description	Function	Fold change	p-value	Ref.
**BMI-1**	B cell-specific Moloney murine leukemia virus integration site 1; Polycomb Repressor Complex 1	Epigenetic repression, chromatin remodeling, modification of hystones	−1,1053	0,0337587	[29, 30]
**GAS41**	Glioma-amplified sequence 41; YEATS domain-containing protein 4	Transcriptional regulation, chromatin remodeling	−1,34902	0,0159932	[31]
**H2AFZ**	H2A Histone family member Z	DNA damage response pathway	−1,10123	0,0098745	[32]
**H1FX**	H1 Histone family member X	Nucleosome assembly	−1,42939	0,0084268	[33]
**SETD1A**	Histone H3-lysine N-methyltransferase	Regulation of chromatin structure, gene expression	−1,12061	0,0112585	[34, 35]
**TBL1XR1**	Transducin (β)-like 1 X-linked receptor 1	Transcriptional regulation	−1,08508	0,0311275	[36, 37]
**SUPT3H**	Suppressor of Ty 3 homolog	Transcriptional regulation	1,11364	0,0493653	[38]

Both *GAS41* and *BMI-1* were down-regulated after Nutlin-3a *in vitro* treatment (fold-change: −1.35 and −1.11, respectively; *p=0.02* and *p=0.03*, respectively).

We further investigated *BMI-1* transcript expression in primary blast cells from Ph^+^ and Ph^−^ ALL patients after *in vitro* treatment with Nutlin-3a observing a trend of reduction in Ph^+^ ALL cells if compared with their untreated counterparts (Figure 6B); instead, Ph^−^ ALL samples showed a different expression of *BMI-1* after treatment (Figure 6C).

## DISCUSSION

P53 pathway is negatively regulated by the interaction between p53 and MDM2 proteins [39]. MDM2 is in turn regulated by ARF tumor suppressor; this binding prevents p53 ubiquitination and activate p53 response [40].

It has been recently shown that *ARF* deletions frequently occur in Ph^+^ ALL patients and contribute to resistance to targeted therapy in ALL induced by BCR-ABL1 [16, 41]. Since MDM2-mediated p53 inhibition is a main inhibitory mechanism in tumors retaining wild-type p53, targeting the MDM2-p53 interaction by small molecules, like Nutlin-3a, represents a novel potential therapeutic strategy to reactivate p53 in cancer [42].

In this study we demonstrated that Nutlin-3a induces growth arrest and apoptosis in Ph^+^ and Ph^−^ ALL cells with wild-type p53, in a dose and time dependent manner. No significant changes in cell viability and apoptosis were observed in p53-null and p53-mutated cell lines after incubation with MDM2 inhibitor, confirming that the p53 pathway can be preferentially activated by Nutlin-3a in cells with wild-type p53. Nutlin-3a treatment activated p53-mediated apoptosis mechanisms, inducing the increased expression of proapoptotic proteins and key regulators of cell cycle arrest in ALL cell lines and primary blast cells.

Given the clinical importance of *BCR-ABL1* mutations, we tested the efficacy of Nutlin-3a in primary blast cells isolated from ALL patients with T315I mutation that confers resistance to available TKIs. We observed that Nutlin-3a reduced cell viability, suggesting a potential alternative therapy for patients resistant to TKIs treatment.

To elucidate the clinical power of this class of compound, we evaluated the effect of the combination between Nutlin-3a and the TKIs which are currently used in Ph^+^ ALL therapy. In particular, we evaluated the co-treatment of Nutlin-3a with Imatinib, Nilotinib and Dasatinib, in Ph^+^ cell lines. The strongest effect was found in BV-173 cell line in which the combination between Nutlin-3a and Imatinib or Nilotinib reduced cell viability of about 40% and 30%, respectively, in comparison with the effect of the single TKI treatment. A less effect of about 10%, even so statistically significant, was observed in BV-173 treated with Nutlin-3a and Dasatinib. Thus, Nutlin-3a can potentiate the effects of TKIs treatment. Furthermore, SUP-B15 is less responsive to the drugs combination. In fact, a significant reduction of viability was only observed in the co-exposure of Nutlin-3a and Imatinib, of about 25%.

Gene expression profile of Nutlin-3a sensitive cells allowed us to identify potential biomarkers of Nutlin-3a clinical activity. In particular, we studied BMI-1 protein involved in control of apoptosis and regulation of *CDKN2A* and *p21* genes [43, 44]. BMI-1 expression is markedly reduced in BV-173 sensitive cells and it could be a biomarker of therapeutic response. We also observed, although in a small number of Ph^+^ ALL samples, a trend of reduction of *BMI-1* transcript after *in vitro* treatment with Nutlin-3a. Thus, BMI-1 role needs to be later confirmed in clinical settings in a large cohort of patients.

Finally, our findings provide a strong rationale for further clinical investigation of Nutlin-3a in Ph^+^ and Ph^−^ ALL. Small-molecule MDM2 antagonists, which target the p53-MDM2 interaction leading to p53-mediated apoptosis, may synergize with current TKIs-based therapies in Ph^+^ ALL patients with wild-type p53 and may represent a valuable strategy for TKIs resistant patients. Moreover, Nutlin-3a may be a novel therapeutic agent for Ph^−^ ALL patients with wild-type p53.

## MATERIALS AND METHODS

### Patients

Bone marrow (BM) and/or peripheral blood (PB) samples were obtained from 11 Ph^+^ and 5 Ph^−^ ALL patients, upon written informed consent, according to the Declaration of Helsinki. Primary blast cells were isolated by density gradient centrifugation over Lymphoprep (Nycomed UK, Birmingham). All patients had *TP53* wild-type sequence; two Ph^+^ ALL patients harbored T315I mutation in the BCR-ABL1 kinase domain. Patient characteristics are shown in Table 2.

**Table 2:** Clinical characteristics of ALL patients

UPN	Gender	Age	Sample Type	Lineage	BCR-ABL1 fusion gene	TP53 status	Karyotype
1	F	69	BM	BCP	+	Wt	46,XX,t(9;22)(q34;q11)
2	M	46	BM	BCP	+	Wt	46,XY,t(9;22)(q34;q11)
3	M	78	BM	BCP	+	Wt	46,XY,t(9;22)(q34;q11)
4	M	30	BM	BCP	+	Wt	N/A
5	M	70	BM	BCP	+	Wt	45,XY,-7,t(9;22)(q34;q11)
6	F	62	PB	BCP	+	Wt	46,XX,t(9;22)(q34;q11)/46,XX,del(9)(p13p22),t(9;22)(q34;q11)/46,XX
7	M	69	PB	BCP	+	Wt	49XY,+der(3),del(3)(p11),+4,der(5)del(5)(p11),t(9;22)(q34;q11)
8	M	64	PB	BCP	+	Wt	N/A
9	M	69	BM	BCP	+	Wt	46, XY,t(9;22)(q34;q11)
10	F	79	PB	BCP	−	Wt	45,XX,der(7)t(7;?)(p11;?),del(8)(q22),add(9)(p21),−9,der(16)t(1;16)(q31;p13)
11	F	51	BM	BCP	−	Wt	46,XX
12	F	75	PB	BCP	−	Wt	N/A
13	F	62	BM	BCP	−	Wt	46,XX
14	M	76	BM	BCP	−	Wt	46,XY
15	F	65	BM	BCP	+	N/A	46,XX,t(9;22)(q34;q11)
16	M	72	BM	BCP	+	N/A	46,XY,t(9;22)(q34;q11)

Abbreviations: UPN, unique patient number; BCP: B cell precursor; wt: wild-type; N/A: not applicable.

### Cell lines

Human Ph^+^ (BV-173 and SUP-B15) and Ph^−^ (NALM-6, NALM-19 and REH) ALL cell lines, and Ph^+^ CML cell line (K562) were obtained from DSMZ (Braunschweig, Germany). Ph^+^ cell lines, BV-173, SUP-B15 and K562, were cultured in RPMI 1640 medium supplemented with 20% fetal bovine serum (FBS), 1% penicillin-streptomycin, and 2 mM L-glutamine (Gibco, Life technologies, Carlsbad, CA, USA). Ph^−^ ALL cell lines, NALM-6, NALM-19 and REH, were cultured in RPMI 1640 medium supplemented with 10% FBS, 1% penicillin-streptomycin and 2 mM L-glutamine (Gibco). Cells were grown at 37°C in 5% CO_2_. All the cell lines harbored homozygous *CDKN2A* deletion. BV-173, SUP-B15, NALM-6 and NALM-19 cell lines lacked genetic alteration of *TP53*. REH cells harbored heterozygous substitution of C>T at position:17:7578389 at codon 181 (R181C) causing functional inactivation of p53 protein; K562 cells were p53-null.

### Reagents and cell treatment

The small-molecule MDM2 inhibitor, Nutlin-3a, was dissolved in DMSO (10 mM stock solution) and stored at −80°C. ALL cells were exposed to increasing concentration of Nutlin-3a (0.5 to 5 μM) for the indicated times. Control cells were treated with DMSO 0.1%. Cells were treated with TKIs at the following concentrations: 50 nM, 100 nM and 250 nM Imatinib, 25 nM, 50 nM and 100 nM Nilotinib, 10 nM, 100 nM, 500 nM Dasatinib.

### RNA extraction

Total RNA was extracted using the RNA Blood Mini Kit (Qiagen GmbH, Hilden, Germany) from mononuclear cells isolated from BM and PB samples. RNA was quantified using the Nanodrop Spectrophotometer and quality was assessed using the Nanodrop and by agarose gel electrophoresis.

### Cell viability assay

Cell viability was measured by colorimetric Methanethiosulfonate (MTS) test (Promega, Inc., Madison, WI). Cells were cultured in 96-well plates at 5×10^4^ cells/100μl with different drug concentrations at 37°C. MTS (0.33 mg/ml per well) was added to each well and the cells were incubated for an additional 3 hours. Following incubation, the optical density was read at 490 nm wavelength. Cellular viability was calculated as percentage of viable cells compared with control (DMSO 0.1%). All experiments were conducted in triplicate. Primary ALL cells were seeded in 6-well plates at 5×10^5^ cells/mL with increasing concentrations of drug for 24 hours and incubated at 37°C. Cell viability was assessed by counting viable and non-viable cells by Trypan blue dye exclusion method. Cellular viability was calculated as percentage of viable cells compared with control (DMSO 0.1%).

### Western blotting

Cells were lysed in RIPA buffer (50 mM Tris-HCl pH 7.5, 150 mM NaCl, 1% NP-40, 0.5% Na Deoxycholate, 0,1% SDS, 1 mM PMSF, complete Protease Inhibitors Cocktail, Roche). For Western blot analysis, the NuPAGE Electrophoresis System was used, according to the manufacturer's instructions (Life technologies). Equal amount of protein extract was transferred to nitrocellulose membranes. The membranes were blocked with 5% milk at room temperature for 1 hour and incubated over night at 4°C with primary antibodies directed towards actin (1:1000; Sigma Aldrich, St Louis, MO, USA, A2066), p53 (1:500; Cell Signaling Techonology, Inc. MA, USA, #2524), BMI-1 (1:1000, Cell Signaling Techonology, #6964), p21/WAF1/Cip1 (1:500; Millipore, Billerica, MA, USA, 05-345), caspase-7 (1:1000; Cell Signaling Techonology, #9492), followed by incubation with horseradish peroxidase-conjugated secondary antibodies at room temperature for 1 hour (1:2000 anti-mouse, 115-035-003 or 1:2000 anti-rabbit, 111-035-003, Jackson ImmunoResearch Laboratories, INC, PA, USA). Protein bands were visualized using an ECL chemiluminescent detection system (Amersham, IL, USA).

### Caspase 3/7 activity assay

Caspase 3/7 activity was measured with the CellEvent^TM^ caspase 3/7 Green Detection Reagent (Life technologies). On cleavage by activated caspase 3/7, the probe becomes fluorescent and free to bind to DNA. Cells were incubated with 1 μM CellEvent^TM^ caspase 3/7 green detection reagent in complete medium for 30 min at 37°C in the dark. Stained cells were observed by flow cytometry.

### Apoptosis

Flow cytometry was performed to detect apoptotic cells after Nutlin-3a treatment. ALL cells were seeded in 6-well plate at 5×10^5^ cells/mL and treated with increasing concentrations of Nutlin-3a or vehicle control (DMSO 0.1%), for different times. After treatment, cells were washed twice in Annexin V binding buffer (0.1 M Hepes/NaOH pH 7.4, 1.4 M NaCl and 25 mM CaCl_2_), stained with Annexin V-FITC/Propidium Iodide (PI) (BD Biosciences, San Jose, CA, USA) and analyzed by FACSCalibur flow cytometry (BD Biosciences).

### Microarray experiments

Gene expression profiling was performed using Affymetrix GeneChip Human Gene 1.0 ST platform (Affymetrix, Santa Clara, CA, USA). Raw data were normalized by using the RMA algorithm and filtered. Genes differentially expressed were selected by analysis of variance (ANOVA) (p-value threshold=0.05, Partek Genomics Suite). The most significantly involved process networks were defined by GeneGo software.

### BMI-1 mRNA expression level

One μg of total RNA was used to synthesize the first strand cDNA using Transcriptor First strand cDNA Synthesis Kit (Roche). BMI-1 expression was evaluated by quantitative real-time PCR (qRT-PCR), performed on Light Cycler 480 II (Roche) with Taqman assay (Life technologies, Carlsbad, CA, USA) using 50 ng of cDNA at the following conditions: 95°C for 10 min, 40 cycles at 95°C for 10 sec, 60°C for 10 sec, 72°C for 15 sec. Each sample was analyzed in triplicate. Relative mRNA expression values were normalized using *GAPDH* as reference gene and calculated using the E^−ΔΔCp^ method.

### Statistical analysis

Statistical analysis was performed by ANOVA in cell lines viability after Nutlin-3a treatment alone or in combination with TKIs, and apoptosis. Student's t-test was used to assess differences in primary cell viability, in caspase 3/7 activation and in *BMI*-1 expression after Nutlin-3a treatment. A p-value < 0.05 was considered statistically significant.

## Abbreviations

Acute lymphoblastic leukemia: ALL
murine double minute 2: MDM2
Philadelphia: Ph+
bone marrow: BM
peripheral blood: PB
Tyrosine kinase inhibitors: TKIs
fetal bovine serum: FBS
methanethiosulfonate: MTS
propidium iodide: PI
analysis of variance: ANOVA
Imatinib: IM
Nilotinib: Nil
Dasatinib: Das
Growth-arrest specific 1 gene: GAS41
UPN: unique patient number
qRT-PCR: quantitative real-time PCR.

## References

[1] Pui CH, Robison LL, Look AT (2008). Acute lymphoblastic leukaemia. Lancet.

[2] Mullighan CG (2011). New strategies in acute lymphoblastic leukemia: translating advances in genomics into clinical practice. Clin Cancer Res.

[3] Iacobucci I, Papayannidis C, Lonetti A, Ferrari A, Baccarani M, Martinelli G (2012). Cytogenetic and molecular predictors of outcome in acute lymphocytic leukemia: recent developments. Curr Hematol Malig Rep.

[4] Martinelli G, Iacobucci I, Soverini S, Piccaluga PP, Cilloni D, Pane F (2009). New mechanisms of resistance in Philadelphia chromosome acute lymphoblastic leukemia. Expert Rev Hematol.

[5] Khoury MP, Bourdon JC (2010). The isoforms of the p53 protein. Cold Spring Harb Perspect Biol.

[6] Moll UM, Petrenko O (2003). The MDM2-p53 interaction. Mol Cancer Res.

[7] Pei D, Zhang Y, Zheng J (2012). Regulation of p53: a collaboration between Mdm2 and Mdmx. Oncotarget.

[8] Sharpless NE (2005). INK4a/ARF: a multifunctional tumor suppressor locus. Mutat Res.

[9] Chang F, Syrjanen S, Tervahauta A, Syrjanen K (1993). Tumourigenesis associated with the p53 tumour suppressor gene. Br J Cancer.

[10] Vousden KH, Lane DP (2007). p53 in health and disease. Nature Rev Mol Cell Biol.

[11] Hof J, Krentz S, van Schewick C, Korner G, Shalapour S, Rhein P, Karawajew L, Ludwig WD, Seeger K, Henze G, von Stackelberg A, Hagemeier C, Eckert C, Kirschner-Schwabe R (2011). Mutations and deletions of the TP53 gene predict nonresponse to treatment and poor outcome in first relapse of childhood acute lymphoblastic leukemia. J Clinical Oncol.

[12] Saha MN, Qiu L, Chang H (2013). Targeting p53 by small molecules in hematological malignancies. J Hematol Oncol.

[13] Chiaretti S, Brugnoletti F, Tavolaro S, Bonina S, Paoloni F, Marinelli M, Patten N, Bonifacio M, Kropp MG, Sica S, Guarini A, Foa R (2013). TP53 mutations are frequent in adult acute lymphoblastic leukemia cases negative for recurrent fusion genes and correlate with poor response to induction therapy. Haematologica.

[14] Stengel A, Schnittger S, Weissmann S, Kuznia S, Kern W, Kohlmann A, Haferlach T, Haferlach C (2014). TP53 mutations occur in 15. 7% of ALL and are associated with MYC-rearrangement, low hypodiploidy, and a poor prognosis. Blood.

[15] Haferlach T, Kohlmann A, Wieczorek L, Basso G, Kronnie GT, Bene MC, De Vos J, Hernandez JM, Hofmann WK, Mills KI, Gilkes A, Chiaretti S, Shurtleff SA, Kipps TJ, Rassenti LZ, Yeoh AE (2010). Clinical utility of microarray-based gene expression profiling in the diagnosis and subclassification of leukemia: report from the International Microarray Innovations in Leukemia Study Group. J Clinical Oncol.

[16] Iacobucci I, Ferrari A, Lonetti A, Papayannidis C, Paoloni F, Trino S, Storlazzi CT, Ottaviani E, Cattina F, Impera L, Abbenante MC, Vignetti M, Vitale A, Potenza L, Paolini S, Soverini S (2011). CDKN2A/B alterations impair prognosis in adult BCR-ABL1-positive acute lymphoblastic leukemia patients. Clin Cancer Res.

[17] Usvasalo A, Savola S, Raty R, Vettenranta K, Harila-Saari A, Koistinen P, Savolainen ER, Elonen E, Saarinen-Pihkala UM, Knuutila S (2008). CDKN2A deletions in acute lymphoblastic leukemia of adolescents and young adults: an array CGH study. Leuk Res.

[18] Vassilev LT (2004). Small-molecule antagonists of p53-MDM2 binding: research tools and potential therapeutics. Cell Cycle.

[19] Vassilev LT, Vu BT, Graves B, Carvajal D, Podlaski F, Filipovic Z, Kong N, Kammlott U, Lukacs C, Klein C, Fotouhi N, Liu EA (2004). In vivo activation of the p53 pathway by small-molecule antagonists of MDM2. Science.

[20] Korotchkina LG, Leontieva OV, Bukreeva EI, Demidenko ZN, Gudkov AV, Blagosklonny MV (2010). The choice between p53-induced senescence and quiescence is determined in part by the mTOR pathway. Aging (Albany).

[21] Demidenko ZN, Korotchkina LG, Gudkov AV, Blagosklonny MV (2010). Paradoxical suppression of cellular senescence by p53. Proc Natl Acad Sci U S A.

[22] Gu L, Zhu N, Findley HW, Zhou M (2008). MDM2 antagonist nutlin-3 is a potent inducer of apoptosis in pediatric acute lymphoblastic leukemia cells with wild-type p53 and overexpression of MDM2. Leukemia.

[23] Zhu N, Gu L, Li F, Zhou M (2008). Inhibition of the Akt/survivin pathway synergizes the antileukemia effect of nutlin-3 in acute lymphoblastic leukemia cells. Mol Cancer Ther.

[24] Kaindl U, Morak M, Portsmouth C, Mecklenbrauker A, Kauer M, Zeginigg M, Attarbaschi A, Haas OA, Panzer-Grumayer R (2014). Blocking ETV6/RUNX1-induced MDM2 overexpression by Nutlin-3 reactivates p53 signaling in childhood leukemia. Leukemia.

[25] Khoo KH, Verma CS, Lane DP (2014). Drugging the p53 pathway: understanding the route to clinical efficacy. Nat Rev Drug Discov.

[26] Park JH, Roeder RG (2006). GAS41 is required for repression of the p53 tumor suppressor pathway during normal cellular proliferation. Mol Cell Biol.

[27] Silva J, Garcia JM, Pena C, Garcia V, Dominguez G, Suarez D, Camacho FI, Espinosa R, Provencio M, Espana P, Bonilla F (2006). Implication of polycomb members Bmi-1, Mel-18, and Hpc-2 in the regulation of p16INK4a, p14ARF, h-TERT, and c-Myc expression in primary breast carcinomas. Clin Cancer Res.

[28] Wu Z, Min L, Chen D, Hao D, Duan Y, Qiu G, Wang Y (2011). Overexpression of BMI-1 promotes cell growth and resistance to cisplatin treatment in osteosarcoma. PloS one.

[29] Bhattacharya R, Mustafi SB, Street M, Dey A, Dwivedi SK (2015). Bmi-1: At the crossroads of physiological and pathological biology. Genes Dis.

[30] Ryland KE, Svoboda LK, Vesely ED, McIntyre JC, Zhang L, Martens JR, Lawlor ER (2015). Polycomb-dependent repression of the potassium channel-encoding gene KCNA5 promotes cancer cell survival under conditions of stress. Oncogene.

[31] Schmitt J, Fischer U, Heisel S, Strickfaden H, Backes C, Ruggieri A, Keller A, Chang P, Meese E (2012). GAS41 Amplification Results in Overexpression of a New Spindle Pole Protein. Genes Chromosomes Cancer.

[32] Brethericka KL, Leacha S, Brooks-Wilson AR (2014). Functional characterization of genetic polymorphisms in the H2AFX distal promoter. Mutat Res.

[33] Shechter D, Nicklay JJ, Chitta RK, Shabanowitz J, Hunt DF, Allis CD, Analysis of Histones in Xenopus laevis (2009). I. A distinct index of enriched variants and modifications exists in each cell type and is remodeled during developmental transitions. J Biol Chem.

[34] Tate CM, Lee JH, Skalnik DG (2010). CXXC finger protein 1 restricts the Setd1A histone H3K4 methyltransferase complex to euchromatin. FEBS J.

[35] Salz T, Li G, Kaye F, Zhou L, Qiu Y, Huang S (2014). hSETD1A Regulates Wnt target genes and controls tumor growth of colorectal cancer cells. Cancer Res.

[36] Li X, Liang W, Liu J, Lin C, Wu S, Song L, Yuan Z (2014). Transducin (β)-like 1 X-linked receptor 1 promotes proliferation and tumorigenicity in human breast cancer via activation of beta-catenin signaling. Breast Cancer Res.

[37] Gonzalez-Aguilar A, Idbaih A, Boisselier B, Habbita N, Rossetto M, Laurenge A, Bruno A, Jouvet A, Polivka M, Adam C, Figarella-Branger D, Miquel C, Vital A, Ghesquieres H, Gressin R, Delwail V, Taillandier L, Chinot O, Soubeyran P, Gyan E, Choquet S, Houillier C, Soussain C, Tanguy ML, Marie Y, Mokhtari K, Hoang-Xuan K (2012). Recurrent mutations of MYD88 and TBL1XR1 in primary central nervous system lymphomas. Clin Cancer Res.

[38] Nakamura Y, Kayano H, Kakegawa E, Miyazaki H, Nagai T, Uchida Y, Ito Y, Wakimoto N, Mori S, Bessho M (2015). Identification of SUPT3H as a novel 8q24/MYC partner in blastic plasmacytoid dendritic cell neoplasm with t(6;8)(p21;q24) translocation. Blood Cancer J.

[39] Lohrum MA, Woods DB, Ludwig RL, Bálint E, Vousden KH (2001). C-Terminal ubiquitination of p53 contributes to nuclear export. Mol Cell Biol.

[40] Manfredi JJ (2010). The Mdm2-p53 relationship evolves: Mdm2 swings both ways as an oncogene and a tumor suppressor. Genes Dev.

[41] Mullighan CG, Williams RT, Downing JR, Sherr CJ (2008). Failure of CDKN2A/B (INK4A/B-ARF)-mediated tumor suppression and resistance to targeted therapy in acute lymphoblastic leukemia induced by BCR-ABL. Genes Dev.

[42] Shangary S, Wang S (2008). Targeting the MDM2-p53 interaction for cancer therapy. Clin Cancer Res.

[43] Huber GF, Albinger-Hegyi A, Soltermann A, Roessle M, Graf N, Haerle SK, Holzmann D, Moch H, Hegyi I (2011). Expression patterns of Bmi-1 and p16 significantly correlate with overall, disease-specific, and recurrence-free survival in oropharyngeal squamous cell carcinoma. Cancer.

[44] Rayess H, Wang MB, Srivatsan ES (2012). Cellular senescence and tumor suppressor gene p16. Int J Cancer.

